# Volar locking plate versus external fixation with optional additional K-wire for treatment of AO type C2/C3 fractures: a retrospective comparative study

**DOI:** 10.1186/s13018-019-1309-4

**Published:** 2019-08-27

**Authors:** Xiaofei Yu, Yadong Yu, Xinzhong Shao, Yanbin Bai, Tong Zhou

**Affiliations:** 1 Department of Hand Surgery, The Third Hospital of Hebei Medical University, NO.139 Ziqiang Road, Shijiazhuang, 050051 Hebei People’s Republic of China; 2 Key Laboratory of Biomechanics of Hebei Province, Shijiazhuang, 050051 Hebei People’s Republic of China

**Keywords:** Unstable intra-articular fracture, Distal radius, Clinical outcome, Volar plate fixation, External fixation

## Abstract

**Purpose:**

This study aimed to compare the radiographic and functional results of *Arbeitsgemeinschaftfür Osteosynthesefragen* (AO) type C2/C3 fracture of distal radius between volar locking plate (VLP) and external fixation (EF).

**Methods:**

It was a retrospective comparative study. Between January 2015 and March 2018, a total of 62/117 patients who underwent EF (23) or VLP fixation (39) for AO type C2/C3 distal radius fractures were assessed. The follow-up period was at least 12 months. Gartland–Werley scale and the disabilities of the arm, shoulder, and hand (DASH) scale were used to evaluate the overall functional outcomes; wrist range of motion and grip strength were measured. The radiographic parameters included radial inclination, volar tilt, radial length, ulnar variance, and articular step-off. All of the comparisons were performed using SPSS 21.0.

**Results:**

The mean follow-up time was 17.1 months. At final visit, VLP performed better in wrist flexion (69.7° vs 62.3°, *p* < 0.001), forearm pronation (73.1° vs 64.8°, *p* = 0.027) and supination (70.6° vs 63.1°, *p* = 0.033) than EF, but not different with regard to other kinematic parameters. No significant difference was found between two groups, in term of Gartland-Werley or DASH score (*p* > 0.05). The ulnar variance and articular step-off was significantly more improved in VLP than EF group, being 0.6 vs 1.6 mm (*p* = 0.002) and 0.5 vs 1.2 mm (*p* = 0.007). The overall rate of complications did not differ in both groups (28.2% vs 34.5%) (*p* = 0.587).

**Conclusions:**

Compared to EF, VLP fixation showed better performance in wrist mobility, correction of ulnar variance, and improving articular congruence, but with the comparable overall functional outcomes and complication rate.

## Introduction

Distal radius fracture is the most common fracture type in the department of orthopedics or emergency, and over 40% of them involved the articular surface [1]. *Arbeitsgemeinschaftfür Osteosynthesefragen* (AO) type C2/C3 distal radius fracture is an unstable completely intra-articular fracture with metaphyseal simple or multifragmentary, which is typically indicated for surgical treatment. Evidences have shown that articular step-off more than 2 mm could increase risk of traumatic arthritis by over four times [2, 3]; radial shortening caused the increased pressure in the distal radioulnar joint [4], and a dorsal angulation over 20° beyond the original position resulted in a transfer of loading across the radioscaphoid and the ulnocarpal joints [5, 6].

During the past 10 years, volar locking plate (VLP) has gained the most popularity in the treatment of distal radius fractures, due to its superior biomechanical property [7, 8]. By contrast, external fixation (EF) is not so extensively used, but was preferred by a fair number of surgeons due to its easy application, improved reduction by ligamentotaxis, no need of secondary procedure, and the acceptable results. However, the higher complication rate should be a concern, including pin-tract infection, loss of reduction, the radial sensory nerve injury, and complex regional pain syndrome [9–11].

Randomized controlled trials (RCTs) or cohort studies have demonstrated the advantages of VLP over EF for treatment of overall types of distal fractures, especially at early postoperative period [12–14]. As for AO type C2/C3 fractures, the reported results were varied and even contradictory, either treated by VLP or EF alone [3, 15, 16], or combined [5, 17]. However, as far as we know, data on the direct comparison of clinical or radiographic outcomes for treatment of such fractures were scarce [18, 19]. The study aimed to compare the EF and the VLP fixation for treatment of AO type C2/C3 distal radius fractures, in terms of radiological outcomes, functional outcomes, and complications.

## Methods

This was a retrospective study, approved by the ethics committee board of The Third Hospital of Hebei Medical University. Inclusion criteria were as follows: age of 18 years or older, definite diagnosis of AO type C2 or C3 fracture, fresh fracture (< 14 days from fracture occurrence), no prior surgery at the injured wrist, unilateral fracture, no concomitant fracture at the injured limb, treatment by VLP or EF, and complete follow-up data available. Exclusion criteria were old fracture, systematic skeletal diseases (e.g., hyperparathyroidism) or local disorder (e.g., tumors, Paget disease, or rheumatoid arthritis), treatment other than VLP or EF, patients lost to follow-up, or incomplete data.

### Surgical technique

#### External fixation

**Fig. 1:**
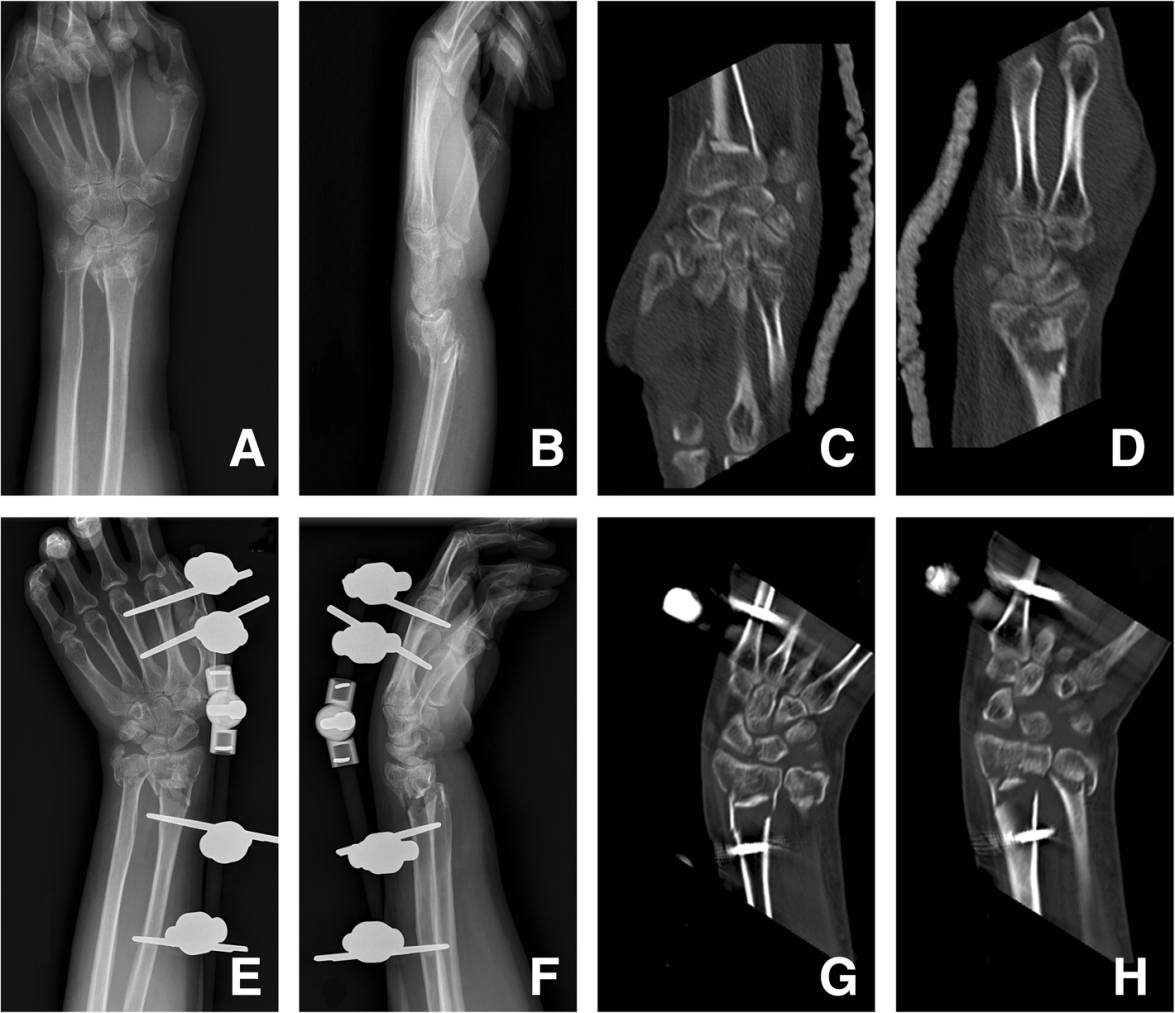
AO C3.1 fracture of the left distal radius occurred in a 52-year-old woman when she fell from standing height. **a**, **b** The preoperative posteroanterior and lateral X-ray. **c**, **d** CT scan showed the articular involvement with multi-fragments. **e**–**h** The good reduction and fixation by EF, from a view of X-ray and CT scanning

After the initial reduction maneuver, continuous slight traction was applied to maintain the reduction and alignment. The external fixator (Zengli Medical Instrument Corporation, Hengshui, China) was fixed on the radius with 4-mm Schanz pins and the second or third metacarpal bone with 3-mm pins (Fig. 1). For fractures that were unsatisfactorily reduced or with the significant articular surface collapse or significant displacement of the larger fragment, a small incision was made at the volar side of the distal radius and the periosteum elevator was introduced to elevate the collapsed fragments under direct vision. In cases of bone detect or seriously impacted fragments, autogenous bone graft or allograft was applied. In most cases, additional average of three K-wires, generally two from the radial side and one from the ulnar side, were used for additional stability.

In the early postoperative period (1–3 days), functional exercises of shoulder, elbow, and finger joints were started to prevent joint stiffness. At 6 weeks after surgery, K-wires and the external fixator were removed and wrist exercises were started. Routine X-ray was taken at the postoperative 3 weeks, 6 weeks, and 3 months to evaluate the status of bone union.

#### VLP fixation

**Fig. 2:**
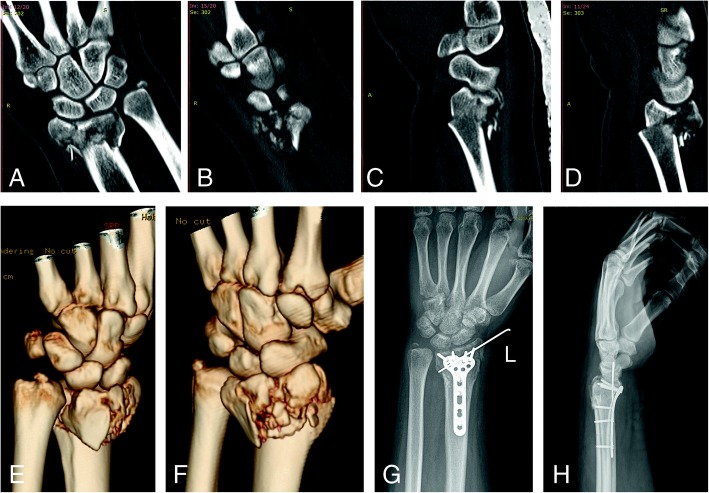
AO C3.1 fracture of the left distal radius occurred in a 37-year-old man due to motor vehicle collision injury. **a**–**f** Preoperative definite diagnosis of comminuted intra-articular fracture of the distal radius, by CT scanning and 3D reconstruction. **g**, **h** The satisfactory reduction and VLP fixation, in posteroanterior and lateral X-ray

Under local or general anesthesia and control of tourniquet in supine position, modified Henry approach was used to make a 10–12-cm longitudinal incision along the course of the flexor carpi radialis (FCR). FCR tendon, the flexor pollicis longus tendon, and radial nerve were retracted ulnarly, and brachioradialis and radial blood vessels were retracted radially. Then, pronator quadratus muscle was elevated from its radial origin and retracted ulnarly to expose the fracture fragments. Every fragment was reduced and re-confirmed under the fluoroscopic guidance. As for impacted fragments into the articular surface or metaphysis, periosteum elevator is introduced to elevate the fragments. Autogenous bone graft or allograft was applied to fill the bone detect, if necessary. Temporary fixation with K-wires was used to stabilize the reduced fragments. 2.4 mm or 3.5 mm T-shape locking plate (Synthes™, Shanghai, China; Wego™, Shandong, China; Trauson Medical Instrument Corporation, Jiangsu, China) and screws were placed, with additional K-wires for auxiliary fixation when necessary (Fig. 2). Postoperatively, cast immobilization was applied for 4 weeks. Early motion of the finger, elbow, and shoulder was started on the first postoperative day. At the 15th day, dressings and sutures were removed. At the 29th day, cast and auxiliary K-wires were removed; active and passive wrist rehabilitation begun and gradually strengthened.

#### Follow-up and postoperative evaluation

The minimum follow-up period was 12 months. The objective functional outcomes were wrist motion range and grip strength. A goniometer was used to measure the wrist flexion, extension, supination, and pronation; a Jamar dynamometer (Jamar, Preston, USA) was used to measure the grip strength. All of these measurements were assessed in comparison with the contralateral uninjured wrist, using percentage as the indicator. The patient-reported DASH score system [20] and the physician-based Gartland–Werley scale [21] were used to indicate the overall functional outcome. DASH questionnaire was used to evaluate patients’ ability to perform the daily activities, with a score range from 0, representing no disability, to 100 points representing maximum disability (the higher the score, the more severe the disability). Gartland–Werley scale is a validated physician-based scoring system, which combines residual deformity, subjective findings, the ROM, the postoperative complications, and poor finger function. The scale ranges from 0 to 52 points, with a higher score representing poorer outcome.

Standard posteroanterior and lateral radiographs were used to measure volar tilt, radial inclination, radial length, ulnar variance, and articular step-off. The Jupiter criteria were used to judge the presence of arthritic changes on the final radiographs [22]. At each visit (postoperative 2 weeks, 6 weeks, 3 months, 6 months, and 12 months), any potential complication was evaluated and documented, either from patients’ self-reports or surgeons’ check-up, including infection, plate/screw loosening, neuropathy or nerve injury, tendon-related issues, loss of reduction, chronic regional pain syndrome, malunion, nonunion, re-fracture, and others.

### Statistical analysis

For comparison of continuous variables (age, surgical duration, volar tilt, radial inclination, radial length, ulnar variance, and articular step-off), Student’s *t* test or Mann–Whitney *U* test was used, based on their data distribution status. As for categorical variables (gender, handedness, injury mechanism, complications), Pearson chi-square test or Fisher’s exact test was used, if appropriate. A *p* value < 0.05 was considered as statistically significant. All the analyses were performed using the SPSS 21.0 software (IBM, Armonk, NY, USA).

## Results

One hundred and seventeen AO type C2/C3 distal fractures were treated surgically in 95 patients, from January 2015 to March 2018. Fifty-one patients with 53 fractures were excluded according to our criteria, with 62 fractures included for data analysis. The injury mechanism was as follows: fall from standing height (23), motor vehicle collision (14), sports-related injury (7), fall from greater height (6), industrial machinery injury (4), and others (8). Thirty-seven patients had involvement of the right wrist and 25 patients had involvement of the left wrist. Eight (12.9%) patient had concomitant ulnar styloid fracture.

In the VLP group, there were 23 men and 16 women and their average age was 52.0 years. In the EF group, there were 13 men and 10 women, with an average age of 50.5 years. There was no significant difference between both groups with respect to age, gender, side of injured wrist, handedness, injury mechanism, time to operation, bone graft, and duration of follow-up (*p* > 0.05)**.** The significantly more operative time (105 min vs 92 min, *p* = 0.023) and greater proportion of fractures requiring K-wire auxiliary fixation (73.9% vs 33.3%) were found in the VLP group, compared to the EF group. **(**Table 1**).**

**Table 1:** Descriptive comparison of perioperative variables between the EF and VLP group

Variable	External fixation		Volar locking plate		*p*
	*n*, % or mean ± SD		*n*, % or mean ± SD		
Age	50.5 ± 15.2		52.0 ± 14.6		0.372
Gender					0.850
Men	13	56.5	23	59.0	
Women	10	43.5	16	41.0	
Wrist fractured					0.690
Right	13	56.5	20	51.3	
Left	10	43.5	19	48.7	
Handedness					0.495
Dominant	15	65.2	22	56.4	
Non-dominant	8	34.8	17	43.6	
Mechanism					0.380
Fall from standing height	8	34.8	15	38.5	
Motor vehicle collision	7	30.4	7	17.9	
Sports injury	2	8.7	5	12.8	
Fall from height	1	4.3	5	12.8	
Industrial machinery injury	3	13.0	1	2.6	
Others	2	8.7	6	15.4	
Type					0.857
C2	17	73.9	28	71.8	
C3	6	26.1	11	28.2	
Time to operation (days)	4.5 ± 3.3		5.8 ± 4.0		0.114
Surgical duration (min)	92 ± 36		105 ± 45		0.023
K-wire auxiliary fixation	17	73.9	13	33.3	0.002
Bone graft	7	30.4	11	28.2	0.852
Duration of follow-up	19.0 ± 6.0		16.0 ± 6.4		0.518

Satisfactory reduction (defined as dorsal tilt < 10°, volar tilt < 20°, radial inclination > 10°, radial shortening < 2 mm, and articular step-off < 1 mm at the immediate postoperative X-rays) was achieved in all fractures, viewed from the postoperative immediate X-rays.

The mean follow-up was 17.1 months (range, 12 to 47 months). The results showed that VLP performed better in wrist flexion (69.7° vs 62.3°, *p* < 0.001), forearm pronation (73.1° vs 66.8°, *p* = 0.027), and supination (70.6° vs 63.1°, *p* = 0.033) than EF. With respect to other parameters (extension, ulnar deviation, and radial deviation), no significant difference was found (*p* > 0.05) (Table 2).

**Table 2:** Comparison of wrist ROM and grip strength at the last visit

	External fixation		VLP fixation		
	Mean (sd)	% of value on contralat. side	Mean (sd)	% of value on contralat. side	
Flexion (deg)	62.3 (7.7)	89.6	69.7 (9.6)	95.6	< 0.001
Extension (deg)	60.2 (11.8)	92.7	61.1 (10.9)	93.7	0.657
Pronation (deg)	66.8 (9.6)	88.3	73.1 (8.7)	94.6	0.027
Supination (deg)	63.6 (8.9)	87.2	70.6 (10.8)	95.2	0.033
Radial deviation (deg)	19.4 (7.7)	90.4	19.7 (6.8)	91.3	0.798
Ulnar deviation (deg)	29.5 (4.6)	91.8	31.0 (5.7)	94.2	0.276
Grip strength (kg)	25.7 (6.2)	94.0	26.2 (7.4)	96.0	0.893

The mean grip strength of the operated wrist was 96% that of the contralateral uninjured wrist in the VLP group, in comparison with 94% in the EF group (*p* = 0.893) (Table 2).

Regarding radiographic parameters, there is no significant difference for comparison of volar tilt, radial inclination, or radial length (*p* > 0.05). The ulnar variance at the final radiographs in the VLP group was 0.6 ± 1.3 mm, and in the EF group was 1.6 ± 1.8 mm, indicating a significant difference (*p* = 0.002). The radial-carpal articular step-off in the VLP group was significantly less than that in the EF group (0.5 ± 1.1 mm vs 1.2 ± 1.4 mm, *p* = 0.007) (Table 3).

**Table 3:** Comparison of radiographic parameters, functional outcomes, and complications between the two groups at the last visit (> 12 months)

Variable	External fixation	VLP	*p*
Volar tilt (deg)	4.9 (5.3)	5.5 (6.1)	0.317
Radial inclination (deg)	20.8 (3.5)	22.2 (4.1)	0.538
Radial height (mm)	10.8 (1.7)	10.4 (1.6)	0.693
Ulnar variance (mm)	1.6 (1.8)	0.6 (1.3)	0.002
Articular step-off (mm)	1.2 (1.4)	0.5 (1.1)	0.007
DASH (points)	16 (12)	12 (15)	0.162
Gartland–Werley score (points)	3.7 (2.4)	2.5 (2.7)	0.086

VLP group exhibited a better Gartland–Werley score (2.5 ± 2.7 vs 3.7 ± .2.4) than EF group, although the difference did not approach to statistical level (*p* = 0.086). As for DASH, both groups exhibited similar excellent performances, scoring 16 ± 12 in the EF group vs 12 ± 15 points in the VLP group (*p* = 0.162) (Table 3).

In the EF group, 9 complications occurred in 8 patients, indicating the incidence of 34.5% (8/23); the pin infection was the most common complication (3, 13.0%), followed by complex regional pain syndrome (2, 8.7%), fixation failure requiring second operation (1, 4.3%), sensory branch of radial nerve injury (1, 4.3%), reduce loss requiring re-adjustment (1, 4.3%), and traumatic radial-carpal arthritis (grade 1, by Knirk and Jupiter criteria). In the VLP group, 13 complications in 11 patients were noted, including 4 cases of plate/screw issues (plate prominence, screw too long or penetration), 3 cases of carpal tunnel syndrome, 2 cases of tendon rupture or tendonitis, 2 case of scar hypertrophy, 1 case of complex regional pain syndrome type I (CRPS) which required long-term physiotherapy, and 1 case of superficial wound infection which resolved by antibiotic therapy. The overall rate of complications did not differ between both groups (*p* = 0.587), although the difference seemed great (34.8% vs 28.2%) (Table 4).

**Table 4:** Complications in both groups

	External fixation, *n* (%)	VLP, *n* (%)	*p*
Overall complications	9 (100)	13 (100)	0.857
Infection	3 (33.3)	1 (7.7)	
Nerve injury	1 (11.1)	1 (7.7)	
Tendon rupture or tendonitis	0	2 (15.4)	
Fixation issues	1 (11.1)	4 (30.8)	
Reduction loss	1	0	
Complex regional pain syndrome	2 (22.2)	1 (7.7)	
Scar hypertrophy	0	2 (15.4)	
Traumatic radial-carpal arthritis	1 (11.1)	0	
Carpal tunnel syndrome	0	3 (23.1)	

## Discussion

As for unstable intra-articular fractures of the distal radius, different surgical treatments have been proposed, but no one exhibited overwhelming superiority to the others [3, 19, 23–25]. The optimal treatment method of choice is still a controversial issue. In the study, we compared the EF and VLP for treatment of type C2/C3 distal radius fractures and demonstrated the superiority of VLP in maintaining joint stability and articular congruence and improving joint mobility. However, at the final follow-up, the overall complications and the functional score based on DASH or Gartland–Werley scale did not differ significantly (*p* > 0.05).

During the past decade, the use of VLP has gained the most popularity in the treatment of unstable distal radius fractures, due to its advantages. On the one hand, the open manner via volar approach allowed good exposure of fracture fragments for easy manipulation, which was more useful in reducing the compressed or impacted fragments. The fixed-angle and locking screw/hole allowed securing the small fragments and provided better support. By comparison, traction alone in EF might not be effective because ligamentotaxis primarily functioned via strong volar links. This could be used to largely explain the difference of final articular step-off (0.5 mm vs 1.2 mm) and ulnar variance (0.6 mm vs 1.6 mm) between both methods. This result was consistent with the previous reports, where EF or VLP was applied for treatment of AO type C unstable distal radius fractures [26, 27]. In these studies, researchers observed a difference value of 0.8 mm (2.2 mm vs 1.4 mm, − 0.4 mm vs − 1.2 mm) in ulnar variance at the final visit (> 52 weeks). But in the other two randomized studies, no difference was observed, either for articular step-off or ulnar variance [18, 28].

We did not observe a significant difference of volar tilt value at the final follow-up, although VLP was superior to EF in correcting volar angulation and afforded enough support via subchondral distal locking screws for a certainly long term (e.g., 12 months). By comparison, loss in volar angulation would continue after removal of the external fixator, from 0.9° at immediate surgery to 4.2° at 6-month follow-up [29]. It should be noted that the small sample size in both groups (23 and 39) should be a concern because of their inadequate ability to detect the true difference in volar tilt, which likely leads to a type II statistical error. If adequate sample size was provided, the advantages of VLP in correction and maintenance of radiographic parameters would be more statistically prominent.

Most previous studies have demonstrated the advantages of VLP over EF in functional recovery at the early postoperative period (< 3 months) [19, 26, 30]. But as for the mid- or long-term period results, controversies exist. In a retrospective cohort of 115 patients with AO type C2/C3 fractures, Richard et al. [10] demonstrated the better DASH score and more improved pronation/supination arc in VLP group at postoperative 12 months. Williksen et al. [26] conducted a RCT study of 104 AO type C fractures and did not observe the significant difference of DASH score and other functional parameters between the two groups, but only the better wrist supination (90° vs 76°) in the VLP group. In a study of 69 AO type C fractures, wrist and pronation range was significantly more improved in the VLP group, while Gartland–Werley or patient-rated wrist evaluation (PRWE) score was non-significantly different [19]. In this study, VLP showed a significantly better performance in wrist flexion, pronation, and supination (*p* < 0.05). This might be attributed to the facts that VLP fixation allowed an earlier wrist mobilization and maintained improved anatomic parameter until fracture union. We suggested the former one (improved anatomic parameters) should be a more important contributing factor, because in this study, VLP fixation did not maximize the benefit of allowing early-period wrist mobilization (approximately at 29th day) and furthermore prolonged casting after plate fixation did not decrease wrist motion [31].

Despite the advantages, VLP could not be applied in some fracture types, for example, the comminuted very distal fractures or osteoporotic fractures that do not allow screw insertion [32]. In such fractures, K-wireaugmented EF could be a better choice, which more likely yields successful results. The continuous distraction of external fixator, together with additional K-wires to secure the comminuted fragments and bone graft to fill the defect, could provide greater stability. In this study, over 70% of EF was supplemented by K-wires, and 30% of cases required bone graft to fill the bone defect, and indeed, these combinations demonstrated the excellent or good functional and radiographic outcomes [27, 33].

With respect to complications, we did not find a significant difference in the incidence rate, 34.5% in the EF group and 28.2% in the VLP group, both of which was in range of the reported figures [10, 18, 19, 26, 33]. Cao et al. [33] retrospectively reviewed 226 type C3 distal radius fractures treated by external fixator in the elderly patients, and reported a rate of 18.6% (42/226) for overall complications, 10% for loss of reduction, 6.2% for joint stiffness, 2.2% for traumatic arthritis, and 0.5% for pin-tract infection. Richard et al. [10] reported a significantly higher overall rate of complications in the EF group (52.5%, 31/59) than that in the VLP group (25%, 14/56). In a meta-analysis of 9 RCTs, Esposito et al. [34] concluded the significantly higher incidence of overall complications or infection in EF over VLP, but non-significant for re-operation, osteoarthritis, malunion, nerve deficit, complex regional pain syndrome, painful retained hardware requiring removal, carpal tunnel syndrome, stiffness, tendon rupture, or tendonitis. It is often difficult to compare these reported varied figures due to differences in study design, patient characteristics, data collection, and follow-up period. On the other hand, complications from physician reports and patient reports are different, with the former emphasizing check-up related complications while the latter often take symptoms as the major concern. McKay et al. [35] suggested not all suboptimal results should be considered as complications, unless it is attributable to a specifically diagnosed complication; the authors proposed a complication checklist to improve prospective data collection.

There were several limitations to this study. Firstly, the retrospective design was its inherent limitation in accuracy of data collection. Secondly, the procedures in this study were performed by 14 surgeons and the surgical choice was mainly dependent on surgeons’ preference, both of which might affect the outcomes. Thirdly, the patient’s pre-injury baseline radiographic parameters or functional state, potential factors influencing the comparative results and the recovery process, could not be examined. Fourthly, the sample size was small due to the scarcity of this complex injury, which might lead to a type II statistical error when evaluating some variables.

## Conclusion

In summary, VLP fixation demonstrated its better performance in wrist mobility (wrist flexion, pronation, and supination), correction of ulnar variance, and improving articular congruence. As for DASH or Gartland–Werley score, other radiographic or functional parameters, and complications, both fixation methods showed similar results. Future research with better design and large sample is needed to verify our results and explore potential contributors that influence the adverse results or complications.

## Data Availability

All the data will be available upon motivated request to the corresponding author of the present paper.

## Abbreviations

AO: *Arbeitsgemeinschaftfür Osteosynthesefragen*
CRPS: Complex regional pain syndrome
CT: Computed tomography
DASH: Disabilities of the arm, shoulder, and hand scale
EF: External fixation
FCR: Flexor carpi radialis
RCT: Randomized controlled trail
VLP: Volar locking plate
